# The impact of COVID19 on sleep quality among healthcare workers

**DOI:** 10.1192/j.eurpsy.2025.1277

**Published:** 2025-08-26

**Authors:** I. Sellami, A. Abbes, A. Feki, A. Haddar, H. Halweni, S. Baklouti, M. L. Masmoudi, M. Hajjaji, K. Jmal Hammami

**Affiliations:** 1 Occupationnal medecine; 2 Rheumatology, CHU Hedi Chaker; 3 General practice, Medecine university, Sfax, Tunisia

## Abstract

**Introduction:**

Since the start of the pandemic, healthcare workers (HCWs) have faced a range of infectious and psychosocial risks. Contracting SARS-CoV-2 has impacted their physical, mental, and emotional well-being, with sleep also likely being affected.

**Objectives:**

Our study aims to assess the impact of SARS-CoV2 infection on sleep quality in HCWs.

**Methods:**

We conducted a descriptive cross-sectional study among staff at Sfax University Hospital who were infected with SARS-CoV-2 between October 2020 and June 2021, during post-COVID follow-up consultations. A questionnaire was utilized, with the medical section completed by a physician to assess sociodemographic, professional, and clinical characteristics of the infection. A self-administered section evaluated the impact of the infection on sleep quality using the Insomnia Severity Index (ISI).

**Results:**

Our study included 200 healthcare workers with an average age of 42.97 years. Nurses comprised 53.5% of the sample, and 41% of the participants were involved in the care of patients with SARS-CoV-2. Workplace infections accounted for 39% of the cases. At the post-COVID follow-up consultation, 83% reported persistent symptoms. According to the Insomnia Severity Index (ISI), 47.5% had no sleep disturbances, 3.5% had mild insomnia, 26% had moderate insomnia, and 8% had severe insomnia. Additionally, 38% of the staff were dissatisfied with their sleep quality following their SARS-CoV-2 infection.

**Conclusions:**

SARS-CoV-2 infection impacts the sleep of healthcare workers, highlighting the need for strategies to improve sleep quality. Addressing these issues is crucial for maintaining staff well-being and ensuring the quality of care provided.

**Disclosure of Interest:**

None Declared

